# A Cobalt-Free Multi-Principal Elements Alloy with Balanced Mechanical Properties and Exceptional Corrosion Resistance

**DOI:** 10.3390/ma19132724

**Published:** 2026-06-25

**Authors:** Jinhong Deng, Manyu Hua, Yangyang Zheng, Yulong Li, Wei Liu, Jingzhong Fang, Yekun Song, Pengfei Wu

**Affiliations:** 1 College of Mechanical and Vehicle Engineering, Changsha University of Science and Technology, Changsha 410114, China; 2 Beijing Institute of Control Engineering, Beijing 100190, China; 3 College of Materials Science and Engineering, Beijing University of Technology, Beijing 100124, China

**Keywords:** high-entropy alloy, mechanical properties, corrosion properties, passive film

## Abstract

This study investigates the mechanical properties and corrosion behavior of a Co-free Fe_40_Ni_30_Cr_20_V_8_Mo_2_ (at.%) multi-principal elements alloy (MPEA) designed for potential applications in aggressive environments. The alloy exhibits a balanced combination of strength and ductility, with a yield strength of approximately 258 MPa, an ultimate tensile strength of about 647 MPa, and a fracture elongation of around 52%, of which deformation is primarily governed by dislocation-mediated plasticity. In terms of corrosion performance, the alloy demonstrates excellent resistance in chloride-containing environments. Potentiodynamic polarization tests reveal a wide and stable passive region of approximately 1.28 V_SCE_ and a high pitting potential of about 0.975 V_SCE_, indicating exceptional stability of the passive film. Electrochemical impedance spectroscopy (EIS) further confirms the high impedance and protective nature of the surface layer. X-ray photoelectron spectroscopy (XPS) analysis reveals that the superior anti-corrosion property is attributed to the formation of a passive film enriched with protective Cr_2_O_3_ and V, Mo oxides, which collectively construct an effective barrier against chloride-induced attack by reducing donor density. This work provides valuable insights for the development of alternative alloys to replace Co-containing systems in demanding corrosive applications.

## 1. Introduction

The rapid advancement of industries such as aerospace, marine engineering, and energy generation imposes increasingly stringent demands on structural materials, particularly for service in extreme environments characterized by high stress, corrosive media, and thermal loads. Traditional alloys, constrained by their traditional compositional design, increasingly fall short of meeting these multifaceted requirements. The emergence of high-entropy alloys (HEAs), also called “multi-principal elements alloys (MPEAs)”, brought a new direction to design high-performance alloys [1]. MPEAs exhibit an unprecedented synergy among their constituent elements. This unique feature facilitates the integrated design of good mechanical properties, wear resistance, oxidation resistance, high thermal stability, and corrosion resistance capabilities, often mutually exclusive in conventional alloys [2,3,4,5,6,7,8,9,10,11,12,13,14,15]. The defining feature of MPEAs is their profound “cocktail effect”, which enables a synergy among constituent elements that transcends simple property averaging. This intrinsic characteristic positions MPEAs as a highly promising platform for next-generation engineering materials.

Among the vast MPEA family, the CoCrFeNi MPEA system have become a quintessential model due to their stable face-centered cubic (FCC) structure, which provides an excellent foundation for combining high toughness with notable corrosion resistance [16,17,18]. The system’s corrosion performance is largely attributed to the formation of a robust, Cr-rich passive film. However, a critical impediment to the sustainable and large-scale application of this promising system is its reliance on Cobalt (Co). Co is associated with significant drawbacks, including documented bio-toxicity, high cost, and complex recyclability, raising environmental and economic concerns. Consequently, the development of high-performance, Co-free alternatives has emerged as a paramount objective within the field [19].

The strategic substitution of Co with carefully selected elements presents a viable pathway to achieve this goal. Extensive research has identified Molybdenum (Mo) as a particularly potent additive for enhancing corrosion resistance in the CoCrFeNi system, Mo enriching within the passive film, preferentially forming stable, high-valent oxides (e.g., MoO_2_, MoO_3_) that act as a formidable barrier against corrosive ion ingress [20,21,22]. In Mo-modified CoCrFeNi-based MPEAs, the native oxide film and the passive films formed at potentials such as 0.5 V_SCE_ and 0.8 V_SCE_ are primarily composed of Mo and Cr as core components, with Mo being the key element responsible for the improved corrosion resistance, as Mo can form stable high-valent oxides and promotes the densification of the passive film and reduces defects, effectively blocking the penetration of corrosive media. The enrichment of Mo within the film fundamentally strengthens the passivation capability and corrosion resistance, serving as the core driving factor for the superior corrosion performance of this system [23,24]. Vanadium (V) introduces severe lattice distortion due to its large atomic radius mismatch with elements like Iron (Fe), Chromium (Cr), and Nickel (Ni), leading to potent solid-solution strengthening without readily precipitating brittle intermetallic phases in FCC matrices [25,26,27]. This effect can dramatically increase yield strength and hardness while preserving appreciable ductility and strain-hardening capability [26,28]. Furthermore, V exhibits a synergistic interaction with Mo; it can co-partition in the passive film, contributing to a denser, more protective oxide structure and thereby offering an additional boost to corrosion resistance [29,30]. Therefore, the co-addition of Mo and V is theoretically predicted to create a synergistic effect, simultaneously addressing the critical needs for enhanced corrosion protection and mechanical robustness in a Co-free alloy.

Guided by this rationale, the present study is designed to comprehensively investigate the effects of substituting Co with synergistic additions of Mo and V in a novel Co-free MPEA, with a focused inquiry into the resultant corrosion mechanisms. We evaluate its mechanical properties via tensile testing and elucidate deformation mechanisms using scanning electron microscopy (SEM) coupled with energy-dispersive X-ray spectroscopy (EDS) and electron backscatter diffraction (EBSD). Electrochemical corrosion behavior in 3.5 wt.% NaCl solution is examined by electrochemical impedance spectroscopy (EIS), potentiodynamic polarization and Mott–Schottky analysis. The elemental valence states of passive films are characterized by X-ray photoelectron spectroscopy (XPS), enabling a direct correlation between film characteristics and macroscopic performance. This work aims to provide a comprehensive understanding of how Mo and V co-addition governs the microstructures, mechanical behaviors, and corrosion mechanisms of this Co-free MPEA.

## 2. Experiments

### 2.1. Materials Preparation

The Fe_40_Ni_30_Cr_20_V_8_Mo_2_ (at.%) high-entropy alloy ingot was fabricated using vacuum arc melting. The Fe_40_Ni_30_Cr_20_V_8_Mo_2_ (at.%) alloy ingot was prepared by vacuum arc melting using high-purity elemental metals (Fe: 99.9 wt.%, Ni: 99.9 wt.%, Cr: 99.9 wt.%, V: 99.9 wt.%, Mo: 99.9 wt.%, provided by Beijing Yanbang New Materials Co., Ltd. (Beijing, China). The ingot was remelted four times to ensure compositional homogeneity. The chemical compositions of the MPEA and the reference alloy (316L SS) were determined by Inductively Coupled Plasma-Optical Emission Spectrometry (ICP-OES, SPECTRO BLUE SOP, Kleve, Germany), as displayed in Table 1. The as-cast ingot was subsequently rolled at room temperature into plates with a thickness of 10 mm. Disk-shaped specimens with an area of 1 cm^2^ and dog-bone-shaped tensile specimens (with a gauge length of 21 mm and a gauge width of 7.5 mm, and a thickness of 1 mm) were cut from the sheets by electrical discharge machining (EDM). The surface oxide layers of all specimens were removed by SiC abrasive paper. Both the disk and dog-bone specimens were then subjected to heat treatment. They were placed in a pre-heated muffle furnace at 1100 °C for 1 min, followed by quenching. Subsequently, the specimens were polished with SiC paper from 180# to 2000# grit. The disc-shaped specimens were embedded in epoxy resin for electrochemical tests, while the dog-bone specimens were prepared for tensile mechanical property testing.

### 2.2. Tensile Test

Room-temperature uniaxial tensile experiments were performed using an MTS Landmark universal testing system at a fixed strain rate of 1 × 10^−3^ s^−1^ (MTS Systems Corporation, Eden Prairie, MN, USA). Three duplicate specimens were tested per processing condition to ensure result reproducibility. Local strain distribution during tensile deformation was characterized by the digital image correlation (DIC) technique.

### 2.3. Electrochemical Tests

Electrochemical tests, including potentiodynamic polarization, electrochemical impedance spectroscopy (EIS), and Mott–Schottky analysis, were conducted using a standard three-electrode system connected to a CHI electrochemical workstation in a 3.5 wt.% NaCl solution. After the samples reached a stable open circuit potential (OCP), all electrochemical tests were performed. The specimen served as the working electrode, with a Saturated Calomel electrode (SCE) and a platinum sheet acting as the reference and counter electrodes, respectively. All electrochemical tests were performed after the samples reached a stable open circuit potential (OCP). The stable OCP of our alloy was approximately −0.29 V_SCE_, and that of 316L SS was approximately −0.35 V_SCE_. Subsequently, EIS measurements were performed by applying a sinusoidal potential perturbation with an amplitude of 10 mV over a frequency range from 10^−2^ to 10^5^ Hz. The potentiodynamic polarization curve was measured at a scan rate of 1 mV/s, starting from a potential of −0.6 V_SCE_ until the current density reached to10^−3^ A/cm^2^. All electrochemical tests were repeated at least three times to ensure the reliability of the data. At a frequency of 1 kHz, Mott–Schottky plots were obtained using an amplitude signal of 10 mV and a step rate of 25 mV in the cathodic direction.

### 2.4. Microstructural Analysis

For microstructural characterization, specimens cut from tested tensile samples using electrical discharge machining were hot-mounted. The mounted samples were ground with SiC abrasive paper from 400# to 3000# grit and then polished with a colloidal silica suspension. X-ray diffraction (XRD) was employed to identify the initial phase constituents of the MPEA specimens. Measurements were conducted on a D/max 2550 in conjunction with Cu K_α1_ (λ = 1.5418 Å) radiation at 40 kV and 30 mA. Diffraction patterns were recorded over a 2*θ* range of 30–110° with a step size (Δ2*θ*) of 0.02° and a scanning rate of 5°/min. Using backscatter electron imaging (BSEI) and electron backscatter diffraction (EBSD), microstructural characterization of both undeformed and tensile-tested samples (with varying local strains) was carried out in a TESCAN CLARA microscope (TESCAN ORSAY HOLDING a.s., Brno, Czech Republic), operated at 30 kV and 20 kV, respectively, with a step size of 50 nm for EBSD mapping. Corresponding chemical compositions were analyzed using EDS, an EDAX system at an accelerating voltage of 15 kV.

### 2.5. Characterization of Passive Films

On a SHIMADZU/Kratos AXIS SUPRA+ spectrometer (Kratos Analytical Ltd., Manchester, UK), X-ray photoelectron spectroscopy (XPS) was employed to characterize the passive films formed on the alloy surfaces.

Disk-shaped specimens were embedded in epoxy resin, ground with SiC abrasive paper (400# to 2000# grit), mechanically polished to a 1 µm finish, and then rinsed with deionized water and anhydrous ethanol. Prior to XPS analysis, stable passive films were established on the sample surface by potentiostatic polarization at 0.1 V_SCE_ for 3600 s. The obtained spectra were processed using XPSpeak software (version: Fitv4.1). Peak fitting was performed using mixed Gaussian–Lorentzian functions after Shirley background subtraction. The binding energy scale was calibrated against the C1s peak at 284.6 eV.

## 3. Results and Discussion

### 3.1. Microstructural Analysis Prior to Deformation

**Figure 1:**
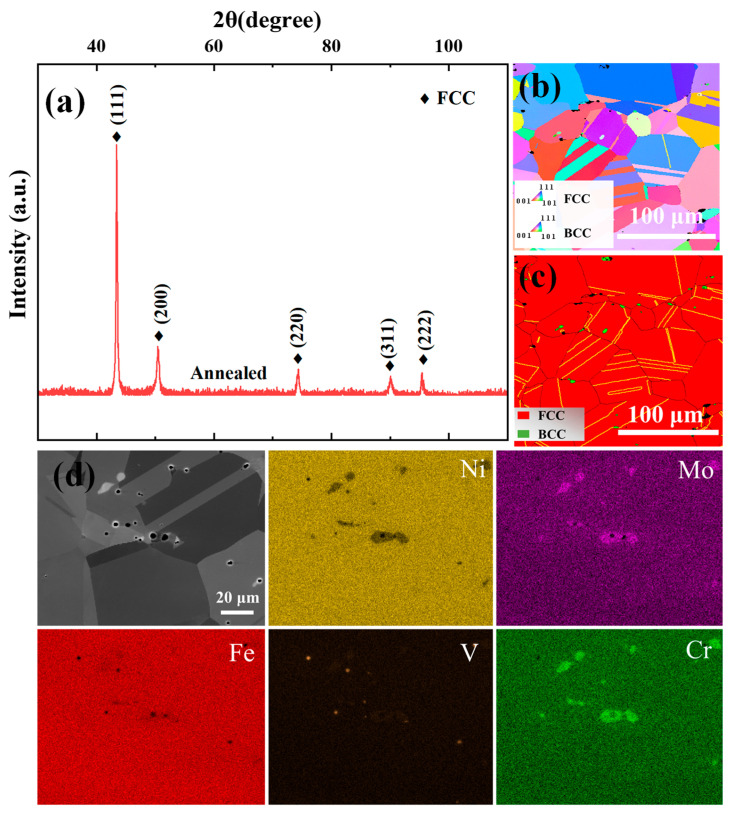
(**a**) XRD patterns of the annealed MPEA; (**b**) EBSD inverse pole figure (IPF) map and (**c**) phase map of the MPEA after heat treatment at 1100 °C; (**d**) EDS elemental maps obtained from the same sample region indicated by the yellow rectangle in (**c**).

Figure 1a shows XRD patterns of the MPEA subjected to annealing. The patterns exhibit five typical FCC peaks, corresponding to the (111), (200), (220), (311), and (222) planes, indicating that the annealed sample predominantly consists of a stable FCC phase. The lattice constant was calculated to be 3.62 Å, which is slightly larger than that of the equiatomic CoCrFeMnNi MPEA (3.56 Å) [31], referring to the lattice strain was enhanced by the addition of V and Mo. Figure 1b,c displays the EBSD IPF image and phase, respectively. Figure 1c is the corresponding EDS elemental mapping of an MPEA sample in Figure 1b. The annealing twins can be easily detected in the IPF map, similar to those observed in the majority of transition MPEAs. However, the phase map (Figure 1b) indicates the presence of a minor BCC phase. The EDS mapping in (c) reveals a relatively uniform distribution of all five constituent elements without significant segregation in the FCC matrix. Analysis suggests the coexistence of the FCC matrix with a small fraction of BCC phase, which may be related to the role of Cr and Mo as a BCC phase stabilizer [29,30]. Due to its low volume fraction, this BCC phase was not detected by XRD. Therefore, the MPEA sample is considered a predominantly single-phase FCC structure.

### 3.2. Mechanical Behaviors

#### 3.2.1. Tensile Deformation Behaviors

**Figure 2:**
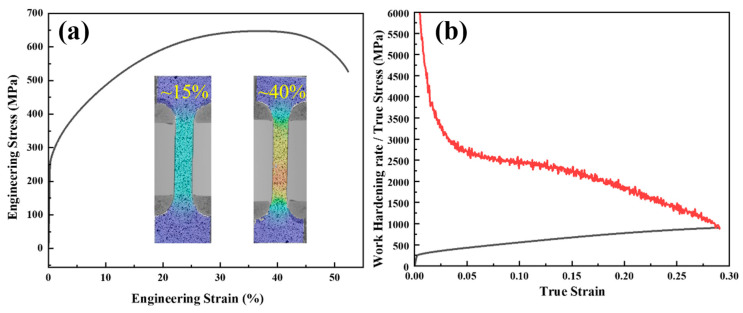
(**a**) Typical engineering stress–strain curve; (**b**) True stress–strain curve and the corresponding work-hardening rate curve of Fe_40_Ni_30_Cr_20_V_8_Mo_2_ MPEA.

Figure 2 displays the mechanical response of the MPEA. The MPEA exhibits a yield strength (YS) of ~258 MPa, an ultimate tensile strength (UTS) of ~647 MPa, and a fracture elongation of ~52%, as shown in Figure 2a. Figure 2b shows the true stress–strain curve and the corresponding work-hardening rate curve of the MPEA. The work-hardening rate generally decreases with increasing true strain. Three distinct stages can be clearly distinguished: a sharp decline in the work-hardening rate at low true strain (ε < 0.025), followed by a plateau-like region with a moderated decrease from 0.025 to 0.15, the rate of decrease moderates, resulting in a relatively flat profile in the curve, and finally a pronounced drop as the true strain exceeds 0.15.

#### 3.2.2. Deformation Microstructures

**Figure 3:**
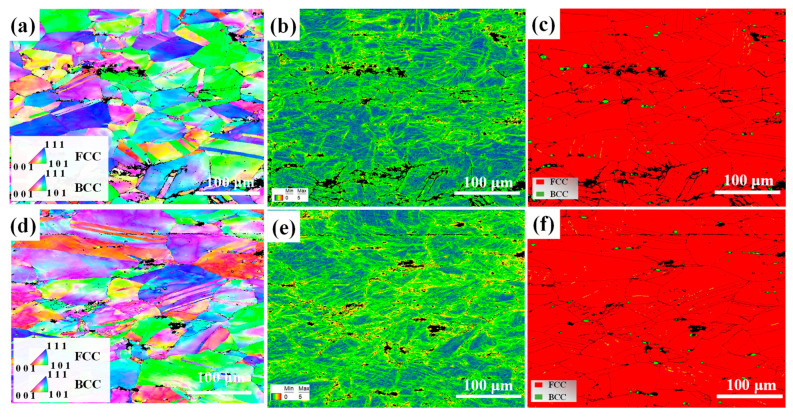
EBSD analysis at different strain levels of the MPEA. (**a**–**c**) correspond to the IPF map, KAM map and phase map at a strain of ~15%, respectively; (**d**–**f**) correspond to the IPF map, KAM map, and phase map at a strain of ~40%, respectively.

Figure 3a–f display the EBSD maps of the MPEA at tensile strains of approximately 15% and 40%, respectively. At ~15%, microstructure is primarily characterized by high-angle grain boundaries and annealing twin boundaries, and grains are almost equiaxed (Figure 3a). Noticeable dislocation accumulation is observed according to Kernel Average Misorientation (KAM) maps (Figure 3b), primarily concentrated at grain boundary regions. In contrast, the IPF map at a strain of ~40% shows that grains are prolonged along the tensile direction (Figure 3d). KAM map shows that grain boundaries and twin boundaries are still the accumulation sites for dislocations, and dislocation density in the grain interior is much higher than that of the 15–strain location (Figure 3e). Throughout the tensile process, no deformation twin structures and martensitic transformation are detected, evidenced by the corresponding phase map (Figure 3c,f), indicating that dislocation activity was the dominant deformation mechanism, which is consistent with XRD results. Notably, what we should focus on is that the KAM value around (Cr, Mo)-rich BCC phase is even higher than that at grain boundaries, indicating that the hard BCC phase led to premature cracking due to stress concentration, deteriorating the elongation.

**Figure 4:**
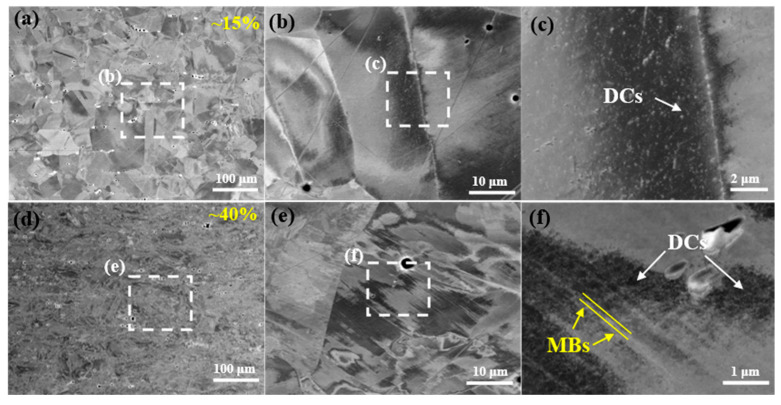
BSE images of sample regions with two representative local strains: (**a**–**c**) microstructures at a strain of ~15%; (**d**–**f**) microstructures at a strain of ~40%. “DCs” and “MBs” denote dislocation cells and microbands, respectively.

The BSE images in Figure 4 provide further insight into the post-deformation microstructures. At the relatively low local strain of ~15% (Figure 4a), the grains undergo slight deformation. While abundant dislocations are generated within the grains, significant dislocation pile-ups are evident (Figure 4c). This observation aligns with the EBSD results, indicating that deformation in the MPEA involves dislocation generation within the grains, thereby influencing its mechanical properties. Under a larger local deformation of ~40%, the grains experience severe plastic deformation, as shown in Figure 4d. The dislocation density within the crystals is considerably higher compared to the region with ~15% strain. Furthermore, numerous microbands are formed at this strain level, as seen in Figure 4e,f. Meanwhile, a few of the dislocation cells with a size of ~200 nm are found within the grains. Normally, dislocation cells are beneficial to ductility by consuming the dislocation-free zones [25]. However, the limited number of dislocation cells cannot provide enough zone to generate abundant dislocations to accommodate plastic deformation. Therefore, this is another reason for the ordinary elongation obtained in the MPEA.

Based on the observed deformation microstructures, the strain-hardening behavior of the MPEA can be elucidated as follows. Initially, upon yielding, the work-hardening rate decreases rapidly (Figure 2b), which corresponds to the activation of dislocation slip and multiplication [26,27]. Subsequently, as strain increases, dislocations accumulate and pile up at grain boundaries, and multiple slip systems become active. The formation of dislocation microbands brings a significant increase in dislocation density, which is reflected in the moderated decline in the work-hardening rate. In the final stage, with further straining, the continued increase in dislocation density and the progressive exhaustion of dislocation-free regions accelerate the decrease in the work-hardening rate until necking.

### 3.3. Corrosion Behaviors

#### 3.3.1. Electrochemical Corrosion Behaviors

**Figure 5:**
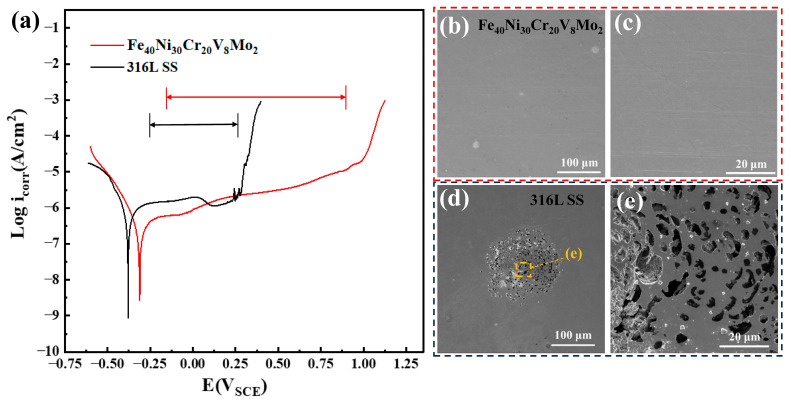
(**a**) Potentiodynamic polarization curves of the MPEA and 316L SS; (**b**–**e**) Corroded surface morphologies of the Fe_40_Ni_30_Cr_20_V_8_Mo_2_ MPEA after polarization within the potential range of −0.5 V_SCE_ to 0.5 V_SCE_.

The potentiodynamic polarization curves of 316L SS and the Fe_40_Ni_30_Cr_20_V_8_Mo_2_ MPEA, measured in a 3.5 wt.% NaCl solution at ambient temperature is presented in Figure 5a. These illustrate the characteristic anodic and cathodic electrochemical responses of both materials. Key quantitative parameters in this plot, including corrosion potential (E*_corr_*), corrosion current density (i*_corr_*), passive current density (i*_pass_*), pitting potential (E*_pit_*), and passivation range (ΔE), were extracted from these curves and listed in Table 2 for comparison.

As a general principle, superior corrosion resistance is associated with a nobler E*_corr_* and a lower i*_corr_* [28]. Combining the data in Table 2 with the polarization curves in Figure 5, it is evident that the Fe_40_Ni_30_Cr_20_V_8_Mo_2_ MPEA possesses a clear advantage over 316L SS. The MPEA exhibits a more noble corrosion potential (E*_corr_* = −0.306 V_SCE_) and a lower corrosion current density (i*_corr_* = 6.423 × 10^−7^ A/cm^2^) compared to 316L SS (E*_corr_* = −0.379 V_SCE_, i*_cor_*_r_ = 5.941 × 10^−6^ A/cm^2^). This convincingly demonstrates the superior corrosion resistance of the MPEA. Furthermore, the results in Figure 5 and Table 2 indicate that the MPEA possesses a broader passivation range and maintains passive stability at higher anodic potentials, suggesting that it can maintain good corrosion resistance even in more severe corrosive environments, thereby exhibiting stronger service adaptability.

In the polarization curve of 316L SS (Figure 5a), a rise in current density occurs at a high potential of 0.028 V_SCE_, which is closely related to the selective dissolution of Cr [32]. Under high potential, the protective Cr^3+^ in the passive film is oxidized to the more soluble Cr^6+^. Compared to Cr^3+^, Cr^6+^ is prone to dissolution and depletion at higher voltage, leading to the breakdown of the passive film integrity and the abnormal change in current density. Furthermore, severe fluctuations in current density are observed in the potential range of 0.216–0.288 V_SCE_ for 316L SS. Within this potential range, the passive film is in a critically unstable state, undergoing a dynamic imbalance between continuous film formation and localized breakdown. Finally, when the potential reaches the pitting potential (E*_pit_* = 0.256 V_SCE_), the film loses its ability to repassivate, marking the onset of stable pitting corrosion.

**Figure 6:**
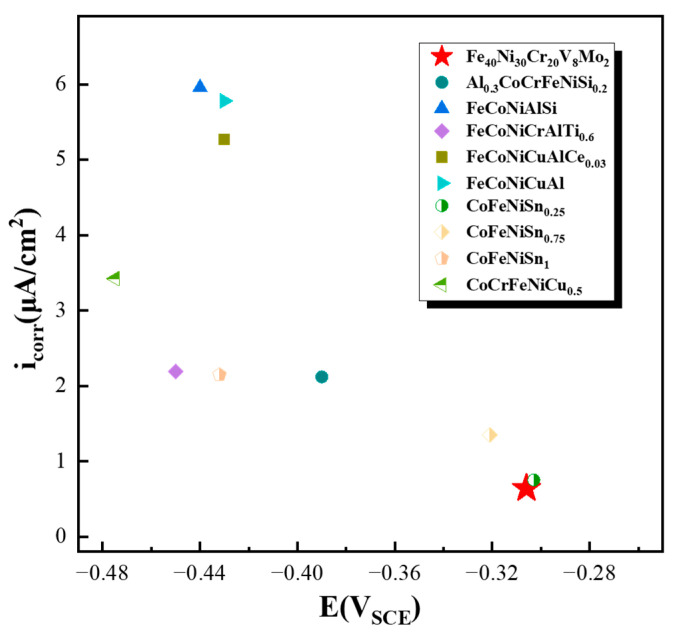
The self-corrosion voltage and currents of the reference and as-prepared MPEAs [33,34,35,36,37,38].

In contrast, the pitting potential (~0.975 V_SCE_) of the Fe_40_Ni_30_Cr_20_V_8_Mo_2_ MPEA is significantly higher than that of 316L SS (E*_pit_* = 0.256 V_SCE_), indicative of a stable and dense passive film formed within its passivation range. In addition, unlike 316L SS, the oxidation of Cr^3+^ to soluble Cr^6+^ is suppressed in the MPEA, likely due to the synergistic effects of its constituent elements. Accordingly, no significant current density fluctuations are observed in the potential interval of 0.884–1.024 V_SCE_, confirming the enhanced stability of the passive film. Moreover, compared to some representative MPEAs [33,34,35,36,37,38], as shown in Figure 6, the alloy in this work possesses a relatively positive E*_corr_* and a lower i*_corr_*, thus superior corrosion resistance is obtained in the present MPEA.

Figure 5b–e show the surface morphologies of the two alloys after polarization within the potential range of −0.5 V_SCE_ to 0.5 V_SCE_. Notably, there are no obvious corrosion pits observed in the surface of the MPEA, as shown in Figure 5b,c. In contrast, Figure 5d,e reveal the distinct pitting traces and corrosion pits of varying sizes on the 316L SS surface. These observations are consistent with the polarization behavior discussed earlier: within this potential window, the passive film of the MPEA remained intact, whereas that of 316L SS underwent localized breakdown. The absence of pitting on the MPEA surface further confirms the superior stability of its passive film and its excellent corrosion resistance, even at elevated potentials. Moreover, as summarized in Table 2, the pitting potential of the MPEA approaches ~1 V_SCE_, suggesting that the alloy is likely to retain its corrosion resistance under even higher applied potentials or in more concentrated aggressive environments.

**Figure 7:**
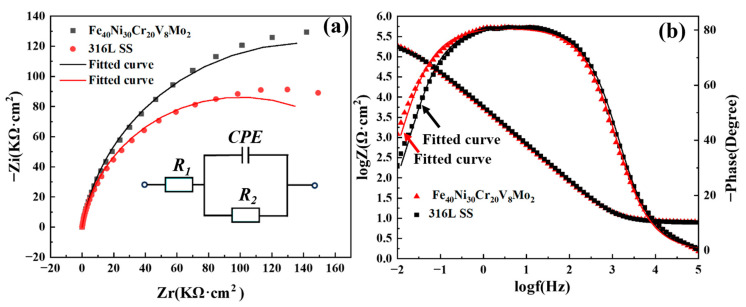
(**a**) Nyquist plots and (**b**) Bode plots for the MPEA and 316L SS in a 3.5 wt.% NaCl solution.

Figure 7 displays the EIS results for the Fe_40_Ni_30_Cr_20_V_8_Mo_2_ MPEA and 316L SS in a 3.5 wt.% NaCl solution, containing Nyquist plots (Figure 7a) and Bode plots (Figure 7b). The capacitive arc radius in the Nyquist plot provides an overview of the charge transfer resistance, providing a comparative assessment of the corrosion resistance of the two materials. The MPEA exhibits a well-defined capacitive arc with a radius significantly larger than that of 316L SS. A larger capacitive arc radius generally indicates that it is harder for the charge transfer process and, consequently, better corrosion resistance [21,28,39]. These observations suggest that the passive film of MPEA demonstrates higher corrosion resistance, which aligns with the findings derived from the potentiodynamic polarization tests discussed earlier.

Figure 7b presents the Bode plots for the MPEA and 316L SS. The magnitude of impedance (|Z|) shows a nearly linear relationship with frequency, with a slope close to −1, indicating capacitive behavior. The curves for both materials largely overlap. In the Bode phase angle plot, notable differences emerge in the low-frequency region (below 0.1 Hz), where the MPEA maintains a higher phase angle than 316L SS, implying enhanced stability and protectiveness of its passive film. In the mid-to-high-frequency region, both materials exhibit similar phase angle values, with a maximum approaching 80°, indicative of a near-ideal capacitive response and the formation of a compact, insulating passive layer.

An R(Q(R)) equivalent circuit model was employed to fit the EIS spectra, with the resulting fitting parameters summarized in Table 3. The fitted chi-squared values were all below 10^−3^, indicating good agreement between the selected EEC model and the experimental data. In these models, R_1_ represents the solution resistance; the values of two systems are equivalent; R_2_ is defined as the charge transfer resistance at the metal/electrolyte interface. To account for non-ideal behaviors arising from surface roughness, material heterogeneity, or current leakage, constant phase elements (CPEs) are introduced in place of ideal capacitors. CPE is parallel to R_2_, indicating the double-layer capacitance here. The MPEA exhibits a significantly higher charge transfer resistance (R_2_ = 2.862 × 10^5^ Ω·cm^2^) compared to 316L SS (2.006 × 10^5^ Ω·cm^2^), indicating superior passivation and corrosion resistance of the MPEA. Additionally, the *n* value of CPE is close to 1, suggesting that this capacitive element behaves nearly like an ideal capacitor, consistent with the formation of a compact and stable passive layer.

**Figure 8:**
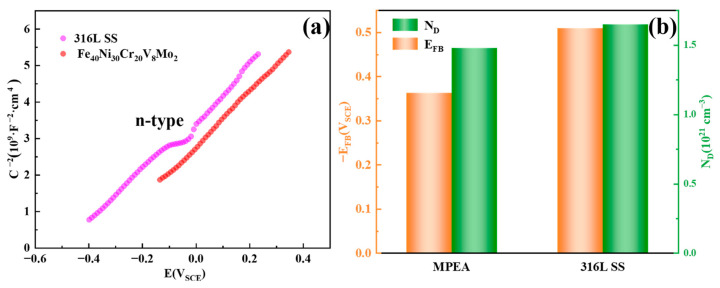
(**a**) Mott–Schottky plots and (**b**) calculated N_D_ and E_FB_ values for the MPEA and 316L SS in a 3.5 wt.% NaCl solution.

As can be seen from Figure 8a, the Mott–Schottky (M-S) curves of both the MPEA and 316L SS exhibit a well-defined linear relationship with a positive slope, indicating that the passive films formed on both materials in 3.5 wt.% NaCl solution presents typical n-type semiconductor characteristics, whose electrical conduction behavior is dominated by donor-type defects (oxygen vacancies and metal interstitial atoms) within the passive films. No distinct negative slope segment is observed within the test potential range, suggesting that the passive films do not show noticeable p-type semiconductor characteristics [40,41,42,43]. From the calculation results shown in Figure 8b, the donor carrier concentration N_D_ of the passive film on the MPEA is significantly lower than that on the 316L SS. The donor carrier concentration is a direct quantitative indicator of the microscopic defect density of the passive film. A lower N_D_ indicates a smaller number of point defects, such as oxygen vacancies in the passive film, corresponding to a denser and more intact passive film. Such a passive film can effectively block the penetration and diffusion of aggressive ions such as Cl^−^, greatly reduce the risk of dissolution and breakdown of the passive film, and thus improve the protection efficiency and long-term stability of the passive film [41,44]. Meanwhile, the flat band potential E_FB_ of the MPEA is significantly positively shifted compared with that of the 316L SS. For n-type semiconductor passive films, a more positive flat band potential corresponds to higher thermodynamic stability of the passive film and a higher potential threshold for pitting initiation, thus endowing the alloy with better pitting resistance and corrosion resistance in chloride-containing environments, which is consistent with the results of the potentiodynamic polarization curves in Figure 5a above.

#### 3.3.2. Characterization of Passive Films

**Figure 9:**
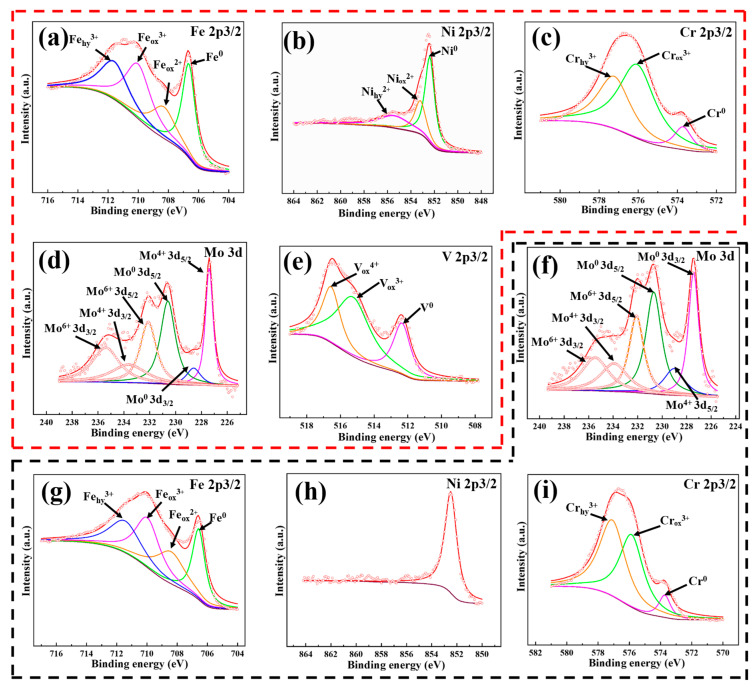
High-resolution XPS spectra after passivation at 0.1 V_SCE_ in 3.5 wt.% NaCl solution: (**a**–**e**) for the MPEA; (**f**–**i**) for 316L SS.

From the electrochemical test results presented above, it is clear that the passive film plays a critical role in determining the corrosion resistance of the alloy. Therefore, XPS was used to characterize the chemical composition of passive films formed on the surfaces of both the MPEA and 316L SS. Figure 9 shows the Cr 2p3/2, Fe 2p3/2, Ni 2p3/2, Mo 3d, and V 2p3/2 XPS spectra collected from the passive films of these two alloys.

In the potentiodynamic polarization curve (Figure 5), the MPEA exhibited a more stable and wider passivation range compared to 316L SS [45,46]. This difference can be attributed to the lower chemical stability of Fe^2+^. Fe^2+^ is prone to dissolution and further oxidation to Fe^3+^, ultimately precipitating as FeOOH on the substrate surface. This “dissolution-reprecipitation” process compromises the denseness and stability of the passive film [47]. As illustrated in Figure 9a,g, the Fe 2p3/2 peak can be resolved into four components, corresponding to Fe^0^ (706.9 eV), Fe^2+^oxide (708.4 eV), Fe^3+^oxide (710 eV), and Fe^3+^hydroxide (711.8 eV) [48]. Notably, the relative intensity of Fe^2+^ in the MPEA’s spectrum (Figure 9a) is lower than that in 316L SS, indicating a higher content of Fe^2+^ in the passive film of 316L SS, which is in agreement with the proposed mechanism.

In contrast, Cr_2_O_3_ possesses high chemical stability within the passive film and is a key protective oxide for enhancing material corrosion resistance [42,49,50]. A higher ratio of Cr_2_O_3_ to Cr(OH)_3_ in the passive film corresponds to superior corrosion performance [51,52]. In the XPS spectra (Figure 9c,i), the Cr 2p3/2 Cr^3+^hydroxide (577.1 eV) [48]. The MPEA’s passive film shows a higher Cr_2_O_3_ to Cr(OH)_3_ ratio compared to that of 316L SS. This directly correlates with its higher pitting potential (E*_pit_*, from Figure 5/Table 2) and charge transfer resistance (R_2_ from Table 3), as a film richer in Cr_2_O_3_ is more effective at blocking the penetration of aggressive chloride ions [53]. The superior corrosion resistance of the MPEA’s passive film is consistent with this finding.

Shown in (Figure 9d,f), the deconvolution results of the high-resolution Mo 3d XPS spectrum demonstrate that Mo element exists in three valence states in the passive film, with their characteristic binding energies as follows: metallic Mo^0^ (3d5/2: 227.80 eV; 3d3/2: 230.97 eV), tetravalent Mo^4+^ (3d5/2: 229.17 eV; 3d3/2: 232.32 eV) and hexavalent Mo^6+^ (3d5/2 232.46 eV; 3d3/2 235.71 eV) [54]. Mo^6+^, among them, mainly exists in the form of soluble molybdates (MoO_4_^2−^). These oxygen-containing molybdate species possess excellent self-healing capability and can rapidly migrate to the damaged regions of the passive film. They cannot only directly block the corrosion channels, but also synergistically promote the reformation of the Cr_2_O_3_ passive layer, further enhancing the long-term protective performance of the passive film [54,55].

In Figure 9e, the V 2p3/2 peak can be deconvoluted into V^0^ (512.5 eV), V^3+^oxide (515.8 eV), and V^4+^oxide (516.9 eV) [56,57]. The incorporation of V into the passive film contributes to an increased overall oxide content, promoting the formation of a denser and thicker passive layer, which in turn enhances the stability and protectiveness of the film [56]. This beneficial effect is reflected in the electrochemical behavior of the MPEA. Unlike 316L SS, which exhibits a distinct current density drop at high potentials attributed to the oxidation of Cr^3+^ to soluble Cr^6+^, no such feature is observed in the polarization curve of the MPEA. This absence is likely associated with the presence of V oxides, which have been reported to suppress Cr segregation and mitigate the destabilization of the passive film [58,59,60,61]. Consequently, the V-containing MPEA maintains a more stable passivation range and a more robust passive film under anodic polarization.

To elucidate the role of Ni in the passive film, the Ni 2p3/2 spectrum of the MPEA in Figure 9b was deconvoluted into three components: Ni^0^ (851.2 eV), Ni^2+^oxide (852.1 eV), and Ni^2+^ hydroxide (857.6 eV) [62]. In contrast, no distinct components were resolved for the Ni 2p3/2 peak of 316L SS in Figure 9h, suggesting a substantially lower Ni content or a different chemical state distribution within its passive layer. Although the oxidation of Ni is thermodynamically less favorable compared to elements such as Cr and Fe, and metallic Ni^0^ tends to enrich at the alloy/film interface [63], distinct Ni oxide species are nevertheless detectable in the passive film of the MPEA. According to the relative peak intensities, Ni^0^ and Ni^2+^ oxide appear to be the predominant forms of Ni present. The incorporation of oxides in the passive film is significant, as Ni oxides enhance the densification and corrosion resistance of the film [30,64]. This aligns well with the higher corrosion resistance of the MPEA indicated by its higher impedance in the EIS Nyquist plot (Figure 7a), further corroborating the superior protectiveness of its passive film. It is worth noting that Cr(OH)_3_ typically exhibits a loose and porous structure, and its limited ability to inhibit chloride ion penetration restricts the denseness and corrosion resistance of the passive film [65]. The presence of Ni oxides in the MPEA’s passive film may help mitigate this limitation by promoting a more compact and stable passive layer.

## 4. Conclusions

In this work, the mechanical properties and corrosion behavior of the Fe_40_Ni_30_Cr_20_V_8_Mo_2_ MPEA were systematically evaluated. The principal findings are summarized as follows:

(1) The alloy displays a balanced combination of strength and ductility. Room-temperature tensile tests yield a yield strength of ~258 MPa, an ultimate tensile strength of ~647 MPa, and a fracture elongation of ~52%. Deformation at room temperature of the MPEA is primarily governed by dislocation-mediated plasticity. The alloy demonstrates acceptable, though not exceptional, tensile ductility.
(2) The alloy exhibits superior corrosion resistance in 3.5 wt.% NaCl solution, characterized by a broad and stable passive region, a high pitting potential (~0.975 V_SCE_), and a low corrosion current density in the potentiodynamic polarization curve. Electrochemical impedance spectroscopy further confirms the high impedance and protective nature of the surface passive film. XPS analysis reveals that this superior performance arises from a high proportion of stable Cr_2_O_3_, together with the synergistic enrichment of V and Mo oxides within the passive film. This oxide architecture effectively impairs localized corrosion initiation, endowing the alloy with outstanding resistance to chloride-induced attack.

## Figures and Tables

**Table 1:** The chemical compositions of the MPEA and 316L SS.

Alloys		Fe	Ni	Cr	Mo	Mn	Si	V
Fe_40_Ni_30_Cr_20_V_8_Mo_2_	wt.%	Bal.	31.81	18.07	3.47	-	-	6.90
	at.%	Bal.	30.57	19.60	2.04	-	-	7.64
316L SS	wt.%	Bal.	9.81	17.20	2.10	2.0	0.10	-
	at.%	Bal.	9.33	18.47	1.22	2.03	0.20	-

**Table 2:** Electrochemical parameters of the two samples derived from Figure 5a.

Samples	*E_corr_* (V)	*i_corr_* (A/cm^2^)	E_pit_ (V)	ΔE (V)
Fe_40_Ni_30_Cr_20_V_8_Mo_2_	−0.306	6.423 × 10^−7^	0.975	1.281
316L SS	−0.379	5.941 × 10^−6^	0.256	0.635

**Table 3:** Fitting parameters of the EEC model for the EIS data shown in Figure 7.

Alloys	R_1_ (Ω⋅cm^2^)	R_2_ (Ω⋅cm^2^)	CPE (Ω^−1^cm^−2^ s^n^)	*n*
Fe_40_Ni_30_Cr_20_V_8_Mo_2_	8.409	2.862 × 10^5^	3.773 × 10^−5^	0.89
316L SS	8.131	2.006 × 10^5^	3.395 × 10^−5^	0.90

## Data Availability

The original contributions presented in this study are included in the article. Further inquiries can be directed to the corresponding authors.

## References

[1] Yeh J.-W., Chen S.-K., Lin S.-J., Gan J.-Y., Chin T.-S., Shun T.-T., Tsau C.-H., Chang S.-Y. (2004). Nanostructured High-Entropy Alloys with Multiple Principal Elements: Novel Alloy Design Concepts and Outcomes. Adv. Eng. Mater..

[2] Wang Z., Zhang S. (2023). Research and Application Progress of High-Entropy Alloys. Coatings.

[3] Yang Y.-F., Hu F., Xia T., Li R.-H., Bai J.-Y., Zhu J.-Q., Xu J.-Y., Zhang G.-F. (2025). High entropy alloys: A review of preparation techniques, properties and industry applications. J. Alloys Compd..

[4] Han L., Maccari F., Souza Filho I.R., Peter N.J., Wei Y., Gault B., Gutfleisch O., Li Z., Raabe D. (2022). A mechanically strong and ductile soft magnet with extremely low coercivity. Nature.

[5] Li Z., Tasan C.C., Pradeep K.G., Raabe D. (2017). A TRIP-assisted dual-phase high-entropy alloy: Grain size and phase fraction effects on deformation behavior. Acta Mater..

[6] Wu P., Zhang Y., Han L., Gan K., Yan D., Wu W., He L., Fu Z., Li Z. (2023). Unexpected sluggish martensitic transformation in a strong and super-ductile high-entropy alloy of ultralow stacking fault energy. Acta Mater..

[7] Wang K., Wang Z., Peng P., Wu X., Liu W., Zhang H., Zhang H., Li Z., Ju J. (2026). Synergistic enhancement of strength and wear resistance in nickel-based superalloy fabricated by oscillating laser directed energy deposition via laser shock peening. J. Mater. Res. Technol..

[8] Li Z., Pradeep K.G., Deng Y., Raabe D., Tasan C.C. (2016). Metastable high-entropy dual-phase alloys overcome the strength–ductility trade-off. Nature.

[9] Ju J., Wang X., Xiao B., Zhu H., Tran N., Shen Z., Zeng X., Wang J., Sun B., Liaw P.K. (2026). Eliminating intergranular oxidation and microstructural instability in chemically complex intermetallic alloys featuring nano-disorder interfaces. Corros. Sci..

[10] Ju J., Yu H., Kong H., Qi D., Peng P., Shen Z., Ding C., Ma S., Gao H., Wang J. (2025). Unveiling oxidation mechanism and microstructural evolution of L-DED Ni_3_Al-based intermetallic alloy at 800 °C via multi-scale characterization and thermodynamic calculations. Nano Mater. Sci..

[11] Tang J., Li Z.-S., Liu X.-B., Chen G.-D., Liu F., Wang S., Meng F.-G., Wang D.-S., Wang K.-M. (2026). Tribology and oxidation properties of graphite/CuNiTiCrNb HEA coatings produced by laser cladding. Carbon.

[12] Li Y., Wang K., Fu H., Guo X., Lin J. (2022). Microstructure and wear resistance of in-situ TiC reinforced AlCoCrFeNi-based coatings by laser cladding. Appl. Surf. Sci..

[13] Wang K., Du D., Liu G., Pu Z., Chang B., Ju J. (2021). A study on the additive manufacturing of a high chromium Nickel-based superalloy by extreme high-speed laser metal deposition. Opt. Laser Technol..

[14] Wang K., Du D., Liu G., Pu Z., Chang B., Ju J. (2020). High-temperature oxidation behaviour of high chromium superalloys additively manufactured by conventional or extreme high-speed laser metal deposition. Corros. Sci..

[15] Gu J., Ju J., Wang R., Li J., Yu H., Wang K. (2022). Effects of Laser Scanning Rate and Ti Content on Wear of Novel Fe-Cr-B-Al-Ti Coating Prepared via Laser Cladding. J. Therm. Spray Technol..

[16] Muangtong P., Rodchanarowan A., Chaysuwan D., Chanlek N., Goodall R. (2020). The corrosion behaviour of CoCrFeNi-x (x = Cu, Al, Sn) high entropy alloy systems in chloride solution. Corros. Sci..

[17] Wu P., Gan K., Yan D., Fu Z., Li Z. (2021). A non-equiatomic FeNiCoCr high-entropy alloy with excellent anti-corrosion performance and strength-ductility synergy. Corros. Sci..

[18] Wu P., Gan K., Yan D., Zhang Y., Li Z. (2026). Overcoming strength-ductility dilemma in non-equiatomic FeNiCoCrSi high-entropy alloys: Effect of Si content on deformation behavior. Mater. Sci. Eng. A.

[19] Dewangan S.K. (2025). Review of Health Hazards in High-Entropy Alloy Processing Under Laboratory Conditions and Risk Assessment Using a Simple Risk Scoring Model. Toxics.

[20] Dai C., Zhao T., Du C., Liu Z., Zhang D. (2020). Effect of molybdenum content on the microstructure and corrosion behavior of FeCoCrNiMox high-entropy alloys. J. Mater. Sci. Technol..

[21] Ruan Y., Yin C., Liu X., Zhang D., Wu J., Zhu Z. (2025). A cobalt-free dual-phase FeCrNiMoSi high-entropy alloy coating with high wear and corrosion resistance compared with FeCoCrNi. J. Alloys Compd..

[22] Wang J., Wen W., Cheng J., Dai L., Li S., Zhang X., Yang Y., Li H., Hou X., Wu B. (2023). Tribocorrosion behavior of high-entropy alloys FeCrNiCoM (M = Al, Mo) in artificial seawater. Corros. Sci..

[23] Wang Z., Liu Z.-X., Jin J., Tang D.-Z., Zhang L. (2023). Selective corrosion mechanism of CoCrFeMoNi high-entropy alloy in the transpassive region based on the passive film characterization by ToF-SIMS. Corros. Sci..

[24] He D., Bi Z., Mi Z., Song Y., Li F., Hou Y., Wang K. (2026). Corrosion behavior of FexCoCrNiMoy (x = 0.5/1; y = 0/0.1) high-entropy alloys: First-principles and experimental study. Appl. Surf. Sci..

[25] Huang R., Zhang L., Amar A., Liaw P.K., Wang T., Li T., Lu Y. (2024). Achieving excellent uniform tensile ductility and strength in dislocation-cell-structured high-entropy alloys. Int. J. Plast..

[26] Gutierrez-Urrutia I., Raabe D. (2011). Dislocation and twin substructure evolution during strain hardening of an Fe–22wt.% Mn–0.6wt.% C TWIP steel observed by electron channeling contrast imaging. Acta Mater..

[27] Wang B., He H., Naeem M., Lan S., Harjo S., Kawasaki T., Nie Y., Kui H.W., Ungár T., Ma D. (2018). Deformation of CoCrFeNi high entropy alloy at large strain. Scr. Mater..

[28] Song Z., Liu J., Li C., Xia X., Geng X. (2026). Microstructure and corrosion performance of laser-clad FeCoCrNiMoCu_0.8_Six eutectic high-entropy alloy coatings: Role of Si modification. Appl. Surf. Sci..

[29] Wang W., Li Z., Han M., Zhang Y., Mu W., Wang N., Zhang W., Weng Z. (2026). CALPHAD-guided design of corrosion-resistant cobalt-based high-entropy alloys with strength-ductility synergy achieved through V, Nb, and Ta alloying. Intermetallics.

[30] Che C., Jiang M., Li A., Kang K., Zhang J., Huang D., Li G. (2025). Effect of Ni content on the corrosion resistance of CoCr_0.8_FeTi_0.4_Nix high entropy alloys in 3.5 wt% NaCl solution. J. Alloys Compd..

[31] Zhang F., Wu Y., Lou H., Zeng Z., Prakapenka V.B., Greenberg E., Ren Y., Yan J., Okasinski J.S., Liu X. (2017). Polymorphism in a high-entropy alloy. Nat. Commun..

[32] Choundraj J.D., Kelly R.G., Monikandan R., Singh P.M., Kacher J. (2023). Influence of native oxide film on corrosion behavior of additively manufactured stainless steel 316L. Corros. Sci..

[33] Zhao H.-W., Guo Y.F., Duan C.S., Zhang J.-D., Zhang L., Ma H.Z. (2025). Effect of Si content on the wear and corrosion performance of spark plasma sintered Al_0.3_CoCrFeNi high-entropy alloys. Mater. Today Commun..

[34] Babilas R., Łoński W., Boryło P., Kądziołka-Gaweł M., Gębara P., Radoń A. (2020). The influence of cooling rate, chromium and silicon addition on the structure and properties of AlCoCrFeNiSi high entropy alloys. J. Magn. Magn. Mater..

[35] Liu J., Liu H., Chen P., Hao J. (2019). Microstructural characterization and corrosion behaviour of AlCoCrFeNiTix high-entropy alloy coatings fabricated by laser cladding. Surf. Coat. Technol..

[36] Wu Z., Li B., Chen M., Yang Y., Zheng R., Yuan L., Li Z., Tan X., Xu H. (2022). Tailoring magnetic property and corrosion resistance of FeCoNiCuAl high-entropy alloy with Ce additive. J. Alloys Compd..

[37] Weng X., Li W., Ding S., Chen J., He J., Wang W. (2026). Effect of Sn addition on microstructure evolution, corrosion behavior and wear resistance of CoFeNiSn high-entropy alloys. J. Mater. Res. Technol..

[38] Wang Z., Fu J., Feng Y., Wan Y., Tian Q., Chen J., Zhao M., Zhang R., Hou B. (2026). Corrosion behavior and antifouling performance of CoCrFeNiCux high-entropy alloys in 3.5 wt% NaCl solution. Electrochim. Acta.

[39] Wang J., Zhang Z., Dai H., Fujiwara H., Chen X., Ameyama K. (2022). Enhanced corrosion resistance of CoCrFeMnNi high entropy alloy using heterogeneous structure design. Corros. Sci..

[40] Song L., Hu W., Huang S., Guo X. (2023). Electrochemical behavior and passive film properties of Ce-added AlCoCrFeNi_2.1_ eutectic high-entropy alloys in sulfuric acid solution. J. Electroanal. Chem..

[41] Bi D., Chang Y., Luo H., Pan Z., Zhao Q., Cheng H., Wang X., Qiao C., Ni Z., Liu A. (2023). Corrosion behavior and passive film characteristics of AlNbTiZrSix high-entropy alloys in simulated seawater environment. Corros. Sci..

[42] He Z., Dong Y., Tian Y., Wang Y., Zhao D., Yang X., Yang Y., Zhao H. (2025). Effect of Cr content on the microstructure and corrosion resistance of laser cladded FeCoNiMnAl0.5Crx high entropy alloy coatings. J. Alloys Compd..

[43] Huang X., Wang J., Wu Y., Wang Y., Liu P., Chen Z., Zheng S. (2025). A threshold of Cr dominated structure transition and stability enhancement of passive films in high entropy alloy CrxMnFeCoNi. Corros. Sci..

[44] Zheng J., Chen T., Chen H. (2018). Antibiotic resistome promotion in drinking water during biological activated carbon treatment: Is it influenced by quorum sensing?. Sci. Total Environ..

[45] Xia Y., Zhou S., Liang Z., Huang X., Zhao Q., Huang H., Zhao H., Zhan Y., Wang C., Xie Y. (2025). Exploring the influence of alloying elements (Zr, Si, and V) on the corrosion behavior of FeCoNiCr-based high entropy alloys in supercritical water environment. J. Nucl. Mater..

[46] Jiao Y., Zheng W., Guzonas D.A., Cook W.G., Kish J.R. (2015). Effect of thermal treatment on the corrosion resistance of Type 316L stainless steel exposed in supercritical water. J. Nucl. Mater..

[47] Wang D., Lan A., Yang H., Jin X., Qiao J. (2025). Corrosion and passivation behavior of Fe_40_Mn_20_Cr_20_Ni_20_ high-entropy alloys in artificial seawater: Effect of rare earth Ce. Intermetallics.

[48] Wei X., Zhang L., Zhang F., Zhang C., Jia Q., Sun K., Duan D., Jiang H., Li G. (2024). Effect of carbon addition on the microstructure and corrosion resistance of the CoCrFeNi high-entropy alloy. Corros. Sci..

[49] Zengin H., Greul A., Turan M.E., Duchoslav J., Mardare A.I., Hassel A.W. (2025). Corrosion and passivation behaviour of as-cast and heat-treated AlCoCrFeNiX_0.5_ (X = Mo, Ta) high entropy alloys in 3.5 wt.% NaCl solution. Electrochim. Acta.

[50] Jiang Y.Q., Li J., Juan Y.F., Lu Z.J., Jia W.L. (2019). Evolution in microstructure and corrosion behavior of AlCoCrxFeNi high-entropy alloy coatings fabricated by laser cladding. J. Alloys Compd..

[51] Jin X., Yang W., Cui Z., Man C., Ji Y., Cui H. (2026). Electrochemical corrosion and passivation behaviour of laser-melted CoCr Ni_x_Mo_30−x_Nb_6_ + 2% B_4_C high-entropy alloy coatings in acidic and chloride environments. Corros. Sci..

[52] Song Z., Liu J., Li C., Li Y., Xia X., Zhang J. (2025). Investigation on microstructure evolution and corrosion behavior of FeCoCrNiMoCu_x_Si_0.2_ high entropy alloy coating produced by laser cladding. Surf. Coat. Technol..

[53] Khanna R G., Krishnan S., Singh M.K., Rai D.K., Samal S. (2023). A detailed investigation regarding the corrosion and electrocatalytic performance of Fe-Co-Ni-Cr-V high entropy alloy. Electrochim. Acta.

[54] Ge Y., Cheng J., Ma L., Xue L., Zhang B., Hong S., Liang X., Zhang X. (2024). Tailoring microstructure and corrosion behavior of CoNiCrFeMoBSi high-entropy alloy coatings via Mo addition. Surf. Coat. Technol..

[55] Yuan J., Shi Z., Dai L., Ye X., Xu L., Gao J., Liu P. (2026). Environment dependent Ta-Mo synergy toward eutectic high-entropy alloys with strength and tunable corrosion resistance. Intermetallics.

[56] Luo H., Sohn S.S., Lu W., Li L., Li X., Soundararajan C.K., Krieger W., Li Z., Raabe D. (2020). A strong and ductile medium-entropy alloy resists hydrogen embrittlement and corrosion. Nat. Commun..

[57] Han F., Huang X., Li C., Zhang Y., Li C., Wang C. (2026). Galvanic corrosion mechanism regulated by heterogeneous phase in novel V-alloyed CoCrFeNiMn corrosion-resistant high-entropy alloys. Electrochim. Acta.

[58] Shi P., Yu J., Yao B., Si J., Wu L., Wu X., Wang Y. (2024). Uncovering the origin of unique elemental distribution behaviors of Vanadium in high entropy alloys. Int. J. Refract. Met. Hard Mater..

[59] Han F., Li C., Pai Z., Zhang Y., Wang C. (2026). Effect of V content on the microstructure and corrosion behavior of (CoCrFeNiMn)_100−x_V_x_ high entropy alloys. Intermetallics.

[60] Pan B., Xu X., Yang J., Zhan H., Feng L., Long Q., Yao Q., Deng J., Cheng L., Lu Z. (2024). Effect of Nb, Ti, and V on wear resistance and electrochemical corrosion resistance of AlCoCrNiM (M = Nb, Ti, V) high-entropy alloys. Mater. Today Commun..

[61] Esteves L., Christudasjustus J., O’Brien S.P., Witharamage C.S., Darwish A.A., Walunj G., Stack P., Borkar T., Akans R.E., Gupta R.K. (2021). Effect of V content on corrosion behavior of high-energy ball milled AA5083. Corros. Sci..

[62] Yang Y., Dong Y., Liu S., Duan S., Li C., Zhang P. (2024). A novel AlCo_1.2_Cr_0.8_FeNi_2.1_ eutectic high entropy alloy with excellent corrosion resistance. J. Alloys Compd..

[63] Cheng J., Ma Y., Wang X., Cheng L., Cao Z. (2025). Excellent corrosion resistance of Fe_37_Ni_37_Cr_20_Al_3_Ti_3_ medium entropy alloy induced by sequential passivation strategy. J. Alloys Compd..

[64] Katić J., Metikoš–Huković M., Peter R., Petravić M. (2015). The electronic structure of the α–Ni(OH)_2_ films: Influence on the production of the high–performance Ni–catalyst surface. J. Power Sources.

[65] Wang Z., Zhang G.-H., Fan X.-H., Jin J., Zhang L., Du Y.-X. (2022). Corrosion behavior and surface characterization of an equiatomic CoCrFeMoNi high-entropy alloy under various pH conditions. J. Alloys Compd..

